# The Influence of N-Acetyl-selenomethionine on Two RONS-Generating Cancer Cell Lines Compared to N-Acetyl-methionine

**DOI:** 10.3390/cells13110937

**Published:** 2024-05-29

**Authors:** Joachim Greilberger, Philipp Stiegler, Michaela Greilberger, Reinhold Wintersteiger

**Affiliations:** 1 Division of Medicinal Chemistry, Otto Loewi Research, Medical University of Graz, 8010 Graz, Austria; 2 Division of Transplantation Surgery, Medical University of Graz, 8010 Graz, Austria; philipp.stiegler@medunigraz.at; 3 Institut für Laborwissenschaft Dr. Greilberger, Schwarzl Medical Center, 8053 Lassnitzhoehe, Austria; institut@laborwissenschaft.at; 4 Department of Pharmaceutical Chemistry, Institute of Pharmaceutical Sciences, University of Graz, 8010 Graz, Austria; reinhold.wintersteiger@uni-graz.at

**Keywords:** N-acetyl-selenomethionine (NASeLM), N-Acetyl-L-methionine (NALM), T-cell leukemia cells (Jurkat cells), medullary thyroid carcinoma cells (MTC-SK), caspase-3 activity, cell growth, mitochondrial activity

## Abstract

N-acetyl-selenomethionine (NASeLM), a representative of the selenium compounds, failed to convince in clinical studies and cell cultures that it neither inhibits cancer growth nor has a chemoprotective effect. This study aims to find out whether NASeLM shows a growth-inhibiting property compared to the carrier substance N-Acetyl-L-methionine (NALM) on two different cancer cells, namely Jurkat cells and MTC-SK cells. Methods: Jurkat and MTC-SK cells were cultured in the absence or presence of varying concentrations (0–500 µg/mL) of NASeLM and NALM solutions. After 0, 24, 48, and 72 h, mitochondrial activity, cancer cell membrane CP levels, cell growth, and caspase-3 activity were assessed in aliquots of Jurkat and MTC-SK cells. Results: Both substances, NASeLM and NALM, were similarly able to inhibit cell growth and mitochondrial activity of Jurkat cells in a concentration-dependent and time-dependent manner up to 70%. Only the determination of caspase activity showed that only NASeLM was able to increase this to almost 40% compared to the control as well as the same lack of NALM. However, the experiments on MTC-SK cells showed a clear difference in favor of NASeLM compared to NALM. While NASeLM was able to reduce cell growth to up to 55%, the same amount of NALM was only at around 15%, which turned out to be highly significant (*p* < 0.001). The same could also be measured for the reduction in MTC-SK mitochondrial activity. Time dependence could also be recognized: the longer both substances, NASeLM and NALM, were incubated, the higher the effect on cell growth and mitochondrial activity, in favour of NASeLM. Only NASeLM was able to increase caspase-3 activity in MTC-SK cells: at 250 µg/mL NASeLM, caspase-3 activity increased significantly to 28% after 24 and 48 h compared to the control (14%) or the same NALM concentration (14%). After 72 h, this could still increase to 37%. A further increase in the NASeLM concentration did not result in higher caspase-3 activity. Conclusion: NASeLM could clearly increase caspase-3 activity in both cell types, Jurkat or MTC-SK cells, and thus induce cell death. NALM and NASeLM showed a reduction in cell growth and mitochondrial activity in both cell lines: While NALM and NASeLM showed almost identical measurements on Jurkat cells, NASeLM was much more effective on MTC-SK than the non-selenium-containing carrier, indicating that it has additional anti-chemoprotective effects.

## 1. Introduction

Selenium in its various organic and inorganic forms shows great potential as a therapeutic agent alone or in combination in the treatment of cancer. It is known that the organic selenium compounds, in particular, have shown success alongside chemotherapy or radiotherapy and should be used very selectively. However, it is hardly known at what dose and for how long the treatment with selenium should last in order to ensure that there is an antioxidant protective effect, e.g., in glutathione peroxidase, on the one hand and to positively influence the development or cell growth of cancer via other non-apoptotic mechanisms on the other [1,2,3]. Salts, such as sodium selenite, amino acids (Selene-methionine), and the methylated forms (methyl-Selene-cysteine, Selene-methyl-Selene-cysteine) are available beside Selene-methionine-converted Selene-cysteine. Most of them can be turned and metabolized into selenides and incorporated into Selene-proteins [2].

Preclinical studies attribute the strongest pro-apoptotic and anti-proliferative effects on cancer cells to methylated Se-containing compounds such as methyl-selenoic acid, methyl-selenol, and Se-methyl-Se-cysteine [2]. This mechanism appears to be based on the increased activity of selenium-dependent enzymes such as glutathione peroxidase and thioredoxin reductase. The induction of apoptosis in cancer cells occurs through selenium-containing glutathione. Low selenium serum or plasma levels are associated with an increased risk of thyroid [4] and colon rectal cancer [5], while low selenium levels in toenails indicate an increased risk of lung, stomach, and prostate cancer [6,7,8].

It has also been hypothesized that selenium not only reduces the risk of cancer but also its growth and metastasis [9]. However, there is a growing body of scientific evidence suggesting that methionine alone plays an essential role in the growth of cancer cells, with reports of methionine restriction and its influence on antioxidant properties via the glutathione system or polyamine synthesis [10]. It is assumed that cancer cells, which require a high level of energy, also make significant use of the radical mechanism for growth and final differentiation and may also be involved in tumor development and that selenium containing substances is involved in non-apoptosis regulations. Membrane-associated carbonyl proteins are massively oxidatively modified proteins that are produced by radicals. It was able to use this marker to differentiate between cancer cells with high radical activity and low radical activity, which of course also has an influence on therapy options with antioxidants on cells or in the human body. Reactive oxygen and nitrogen species (RONS) develop from free radicals in the presence and absence of metals, as well as through the downregulation of antioxidant enzymatic and non-enzymatic processes in cells, the extracellular matrix, and blood. However, an excess of radicals also causes increased oxidative and nitration damage to healthy organic materials that make up the organism, such as carbohydrates, fats, proteins, and nucleic acids.

In a published study on KARAL infusions, NASeLM and NALM were also used in addition to the two main components, alpha-ketoglutarate and 5-hydroxy-methyl-furfural, which showed an improvement in the quality of life of patients with lung cancer, combined with a reduction in tumor mass, although no general statement can be made due to small numbers, especially in relation to other types of cancer [11]. However, the cellular results confirm this success on a cellular level (work with lung cancer cells). While the cell growth and mitochondrial inhibiting effect of aKG and 5-HMF on cancer cells was confirmed, the explicit effect of NASeLM in the presence of and in contrast to NALM as a chemoprotective substance has not yet been investigated on cell culture like Jurkat or MTC-SK cells. The aim of this study was to investigate whether selenium-containing methionine (NASeLM: N-acetyl-selenomethionine), in contrast to non-selenium-containing methionine (NaLM: Na-acetyl-L-methionine), has an anti-proliferative and growth-inhibiting effect on two different cancer cells with high radical activity, such as Jurkat cells and MTC-SK, as well as increased caspase 3 activity, in a dose-dependent manner.

## 2. Materials and Methods

### 2.1. Materials

Media: RPMI 1640, FBS, DMEM, antibiotic–antimycotic solution (Thermofisher, Vienna, Austria); reagents: Cytofix–Cytoperm permeabilization Kit (Thermofisher, Vienna, Austria), FITC Active Caspase-3 Apoptosis Kit (BD Biosciences Kit; Allschwil, Germany), WST-1 Cell Proliferation Reagent (Abcam; Cambridge, UK); chemicals: NALM and NASeLM (Sigma), guanidine-HCl, butyl-hydroxy-toluene (BHT; Sigma Aldrich, St. Louis, MO, USA), di-nitro-phenyl-hydrazine (DNPH) (Altmann Analytics, Munich, Germany); and flasks (Falco^®^ Cell Culture; Corning Incorporated, New York, NY, USA) were used.

### 2.2. Cell Culture

Jurkat-J6 and MTC-SK were used for cell culture and further experiments. The Jurkat cell line was procured from the National Center for Cell Sciences (Pune, India), and it had a confluency of >80%. It is an immortalized line of human T-lymphocyte cells derived from acute T-cell leukemia. It is used to study acute T-cell leukemia. The Jurkat-J6 cell line has the ability to produce IL-2 and that is why it is useful for different anti-cancer study [12]. It was an immortalized line of human T-lymphocyte cells derived from acute T-cell leukemia. The Jurkat-J6 cell line was centrifuged, re-suspended in complete RPMI-1640 + glutamine media (containing 10% FBS and 1% antibiotic–antimycotic solution) in flasks, and incubated at 37 °C with a continuous 5% CO_2_ supply. Cell density was adjusted to about 1–2 × 10^6^ cells/mL for all of the further experiments.

Medullary thyroid carcinoma cells (MTC-SK) were provided by Prof. Dr. Pfragner from the Institute of Pathophysiology at the Medical University of Graz. In the MTC-SK cell line, the predominant findings were terminal chromosomal rearrangements most frequently concerning chromosome 11p, i.e., the locus of the calcitonin and calcitonin gene-related peptide genes and the H-ras oncogene, and a characteristic instability of the centromeric region of chromosome 16 and somatic pairing of the homologous chromosomes 16 [13].

### 2.3. Cell Proliferation Experiments

Cell proliferation experiments were carried out by incubating different NASeLM or NALM concentrations (0, 125, 200, 250, 500 µg/mL) in an appropriate medium for 24, 48, and 72 h with the cells. The cell proliferation was estimated with the CASY^®^ Cell Counter (Hoffmann-La Roche Ltd., Basel, Switzerland). Aliquots were used for further experiments.

### 2.4. Cytotoxicity Assay

A cytotoxicity assay (WST assay) was carried out to determine the viability all used cancer cell lines as used in our previous article [14,15]. The WST-1 Cell Proliferation Reagent provided a simple and accurate method for measuring cell proliferation. The assay was carried out as described in the manufacturer’s instructions. In brief, cells (200,000/mL) were seeded onto transparent 24-well plates and incubated for 0, 24, 48, and 72 h in the absence or presence of NASeLM or NALM (0, 125, 200, 250, 500 µg/mL). The cells were then washed twice with DPBS 1X and incubated in a fresh medium with 10% WST-1 reagent for 2 h. The absorbance was measured at 450 nm (690 nm was used as a reference wavelength and subtracted) in a spectrophotometric reader (Spectra Max Pro 384; Molecular Devices; San Jose, CA, USA).

### 2.5. Caspase-3 Activity Measurements

After incubation for 72 h in the absence of any substances (negative control), in the presence of 4 or 0.4 µM CPT (positive control), or in the presence of either NASeLM or NALM (0, 125, 200, 250, 500 µg/mL), a population of 1.0 × 10^6^ cells/mL of all used cancer cell lines was centrifuged (350× *g*) and re-suspended in PBS; the supernatant was removed, and the cells were washed again with cold PBS and then centrifuged (350× *g*). After removing the supernatant, the pellet was re-suspended in 500 µL of Cytofix–Cytoperm solution and incubated on ice for 20 min. After the washing and centrifugation steps and the use of 500 µL of Perm/Wash buffer three times, the samples containing 100 µL of Perm/Wash buffer were incubated with 20 µL of antibody–FITC for 30 min in the dark. After incubation, the samples were washed with Perm/Wash buffer and centrifuged (350× *g*); the supernatant was removed, and the samples were re-suspended in the Perm/Wash buffer three times. FITC–Caspase-3 activity was measured on an FACS Calibur^®^. The supernatant was collected, and 50 μL of reaction buffer and 5 μL of DEVD-AFC (final concentration of 50 μM) were added and further incubated for 2 h at 37 °C. Resultant fluorescence was measured by using excitation and emission wavelengths of 495 and 519 nm, respectively.

### 2.6. Statistical Analysis

Group comparisons were made by using *t*-tests where appropriate and indicated. Linear regression and exponential regression curves were calculated based on Pearson’s regression (SPSS 25, SPSS Inc., Chicago, IL, USA). All values are given as mean values and standard deviations. Statistical significance was considered at *p* < 0.01, with high significance at *p* < 0.001.

## 3. Results

### 3.1. Jurkat Cell Lines

#### 3.1.1. Cell Proliferation Experiment

**Figure 1:**
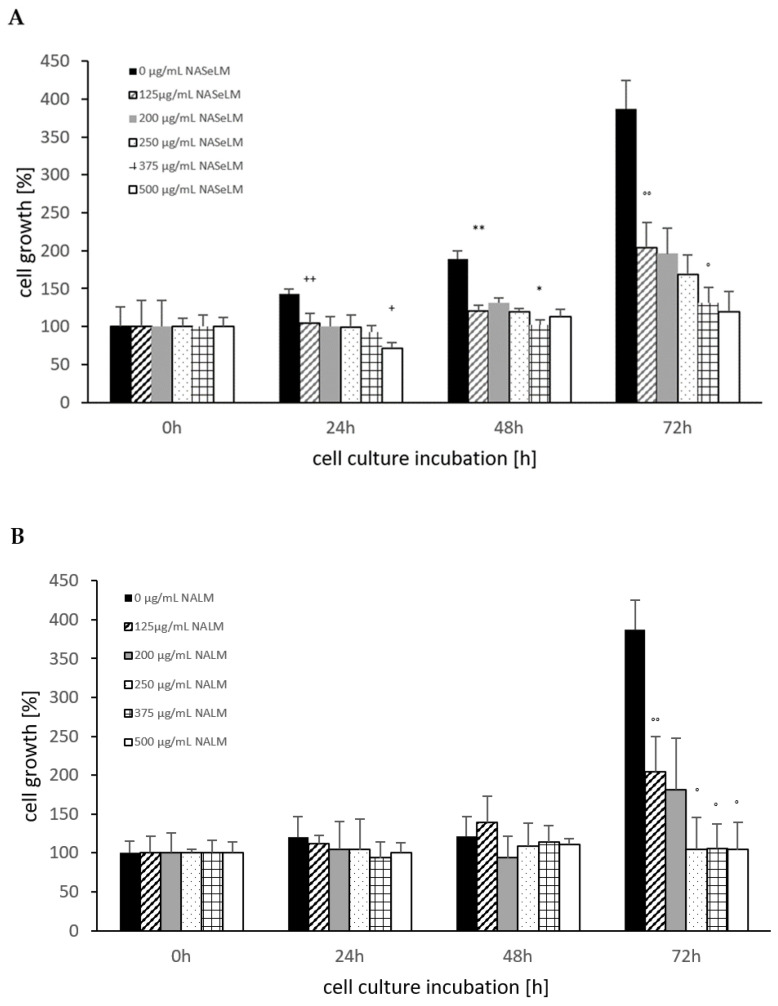
Cell growth on Jurkat cells in absence or presence of different NASeLM (**A**) or NALM (**B**) concentrations after 24, 48, and 72 h. (**A**): ^++^ *p* < 0.01: significance between control and 125 µg/mL NASeLM after 24 h incubation. ^+^ *p* < 0.05: significance between 125 µg/mL NASeLM and 500 µg/mL NASeLM after 24 h incubation. ** *p* < 0.01: significance between control and 125 µg/mL NASeLM after 48 h incubation. * *p* < 0.05: significance between 125 µg/mL NASeLM and 375 µg/mL NASeLM after 48 h incubation. °° *p* < 0.01: significance between control and 125 µg/mL NASeLM after 72 h incubation. ° *p* < 0.05: significance between 125 µg/mL NASeLM and 375 µg/mL NASeLM after 72 h incubation. (**B**): °° *p* < 0.01: significance between control and 125 µg/mL NALM after 72 h incubation. ° *p* < 0.05: significance between 125 µg/mL NASeLM and 250, 375, and 500 µg/mL NALM after 72 h incubation.

The control shows an increase in cell growth of the Jurkat cell line, which has almost doubled after 48 h and tripled after 72 h (Figure 1A). The cell values are shown in percentages. Already after 24 h incubation, a significant reduction was observed with 125 µg/mL NASeLM compared to the control (104.5 ± 16.0%) vs. 143.5 ± 6.0%); n = 3; *p* < 0.01). An increased concentration with 200, 250, and 375 µg/mL NASeLM showed no further inhibition. Only the highest concentration of NASeLM (500 µg/mL; 71.5 ± 7.5%; n = 3; *p* < 0.05) showed a significant reduction in cell growth compared to 125 µg/mL NASeLM. The calculated IC50% was 500 µg/mL NASeLM.

After 48 h of incubation, a higher significant reduction was observed between the control experiment and 125 µg/mL NASeLM (189.5 ± 10.5% vs. 121.0 ± 12.5% n = 3; *p* < 0.01). A further significant reduction was measured between 200 and 375 µg/mL NASeLM (131.0 ± 7.0% vs. 102.5 ± 6.0%; n = 3; *p* < 0.05). The IC50% at 48 h was 375 µg/mL NASeLM.

With the highest incubation time of 72 h, the IC50% could already be achieved with the use of 125 µg/mL NASeLM (204.0 ± 41.0% vs. 387.5 ± 37.3%) with a significance of *p* < 0.01 (n = 3). The other concentrations were able to further reduce growth: 375 µg/mL NASeLM (131.0 ± 21.0%; n = 3) showed a significance of *p* < 0.05 against 125 µg/mL NASeLM and *p* < 0.001 regarding the 72 h control.

NALM was only able to show a reduction in cell growth of the Jurkat cells at the highest incubation time. A concentration of 125 µg/mL NALM caused a significant reduction from 387.5 ± 37.5% to 204.0 ± 46.0% (*p* < 0.01; n = 3). Increasing the concentration to 250 µg/mL caused a decrease in cell growth (104.5 ± 41.0%; n = 3; *p* < 0.05) compared to 125 µg/mL NALM, while no significant reduction in cell growth was achieved at 375 and 500 µg/mL NALM.

When comparing the cell growth data between NALM and NASeLM with the same concentration after 48 and 72 h on Jurkat cells, no significant difference was obtained, indicating that selenium does not play any role in this process, as shown in Table 1.

#### 3.1.2. Mitochondrial Activity of Jurkat Cell Lines in Presence or Absence of NASeLM or NALM

**Figure 2:**
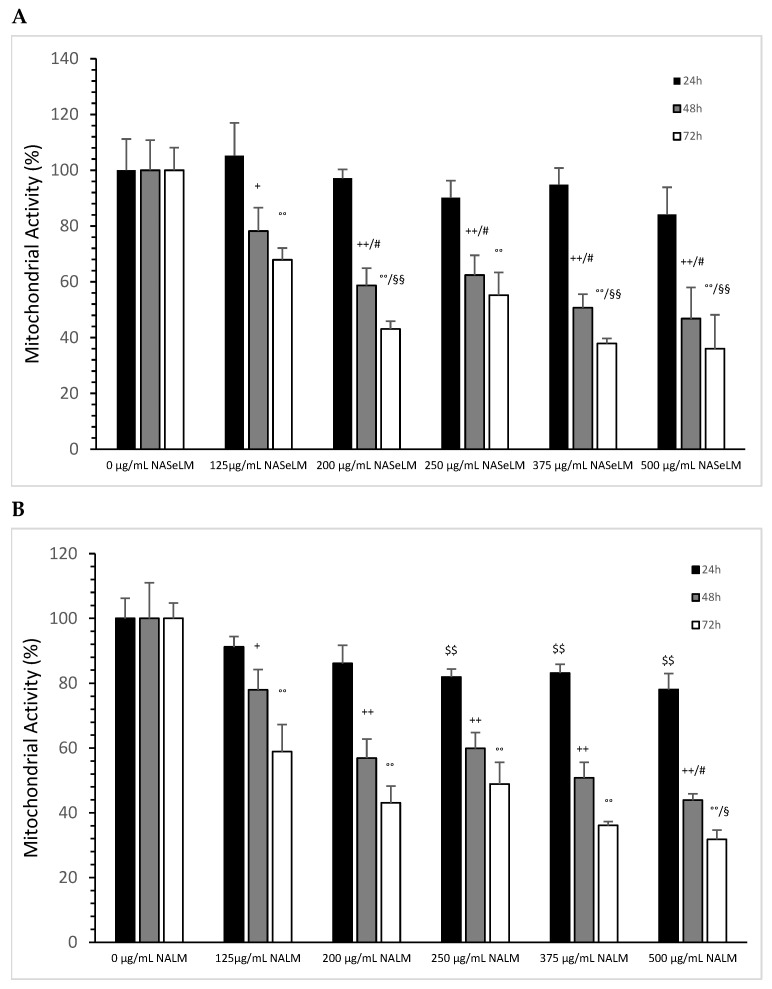
Mitochondrial activity measurements on Jurkat cells in absence or presence of different NASeLM (**A**) or NALM (**B**) concentrations after 24, 48, and 72 h. (**A**): ^+^ *p* < 0.05: significance between control and 125 µg/mL NASeLM after 48 h incubation. ^++^ *p* < 0.01: significance between control and 125, 200, 250, 375, and 500 µg/mL NASeLM after 48 h incubation. ^#^ *p* < 0.05: significance between 125 µg/mL and 200, 250, 375, and 500 µg/mL NASeLM after 48 h incubation. °° *p* < 0.01: significance between control and 125, 200, 250, 375, and 500 µg/mL NASeLM after 72 h incubation. ^§§^ *p* < 0.05: significance between 125 µg/mL NASeLM and 200, 375, and 500 µg/mL NASeLM after 72 h incubation. (**B**): ^$$^ *p* < 0.01: significance between control and 250, 375, and 500 µg/mL NALM after 24 h incubation. ^+^ *p* < 0.05: significance between control and 125 µg/mL NALM after 48 h incubation. ^++^ *p* < 0.01: significance between control and 200, 250, 375, and 500 µg/mL NASeLM after 48 h incubation. ^#^ *p* < 0.05: significance between 250 µg/mL and 375 µg/mL NALM after 48 h incubation. °° *p* < 0.01: significance between control and 125, 200, 250, 375, and 500 µg/mL NALM after 72 h incubation. ^§^ *p* < 0.05: significance between 200 µg/mL and 500 µg/mL NALM after 72 h incubation.

After 24 h of incubation, none of the NASeLM concentrations used showed a significant reduction in mitochondrial activity compared to the 24 h control (Figure 2A). After 48 h of incubation, a concentration-dependent reduction in mitochondrial activity was observed with NASeLM. The use of 125 µg/mL NASeLM showed a significant reduction in mitochondrial activity of 78.2 ± 8.4% compared to the control (100.0 ± 10.8%; n = 3; *p* = 0.05), which was further reduced to 58.7 ± 6.2% at 200 µg/mL NASeLM (*p* < 0.01; n = 3). In addition, significance could also be achieved between 125 µg/mL and 200 µg/mL NASeLM after 48 h of incubation (*p* < 0.05). Increasing the concentration to 250, 375, and 500 µg/mL NASeLM did not decrease mitochondrial activity any further. The IC50% of NASeLM on the mitochondrial activity of Jurkat cells was achieved at a concentration around 375 µg/mL. The inhibition of mitochondrial activity was more pronounced after 72 h compared to 48 h or 24 h. A significance of *p* < 0.01 could already be achieved with 125 µg/mL NASeLM compared to the 72 h control (67.9 ± 4.2% vs. 100.0 ± 8.1%; n = 3). The use of 200 µg/mL NASeLM already showed the most effective inhibition of mitochondrial activity (43.1 ± 2.8% vs. 100.0 ± 8.1%; *p* < 0.001; n = 3). The use of 250, 375, and 500 µg/mL NASeLM did not show any further inhibition of mitochondrial activity after 72 h. The IC50% after 72 h of incubation could be calculated at 175 µg/mL, which was below the IC50% at 48 h.

The experimental trials in the presence of NALM were able to influence mitochondrial activity, as shown in Figure 2B. After 24 h of incubation with 250 µg/mL NALM, there was a significant decrease in mitochondrial activity from 100 ± 6.2% to 81.9 ± 2.5% (*p* < 0.01). Increasing the concentration of NALM to 375 and 500 µg/mL did not cause any further change; the reduction levelled off at 20%. After 48 h of incubation on Jurkat cells, there was already a clear reduction in activity to 78.0 ± 6.2% (n = 3) compared to the control (100 +/− 11.0%; n = 3; *p* < 0.05), and at 200 µg/mL (56.9 ± 5.9%, n = 3), there was a further significant reduction compared to the control (*p* = 0.03). The lowest activity was measured in the presence of 500 µg/mL NALM (43.9 ± 2.0%; n = 3; *p* < 0.05) compared to the 200 µg/mL concentration. The IC50% was determined with 375 µg/mL NALM at 48 h of incubation on Jurkat cells.

After 72 h, all concentrations of NALM used showed a decrease in mitochondrial activity. A significance of *p* < 0.01 was achieved between the control (100 ± 4.8%) and 125 µg/mL NALM (58.9 ± 8.4%; n = 3). The use of 200 µg/mL NALM caused a further significant decrease in activity of 43.1 ± 5.2% (n = 3; *p* = 0.01) compared to 125 µg/mL NALM. The loss of activity was prolonged up to 31.8 ± 2.9% at 500 µg/mL NALM compared to 200 µg/mL (*p* < 0.05).

When comparing the mitochondrial activity between the same concentration of NALM and NASeLM at the same incubation times, no significant difference was found (Table 2).

#### 3.1.3. Caspase-3 Activated Apoptosis Measurements of Jurkat Cell Lines in Presence or Absence of NASeLM or NALM

**Figure 3:**
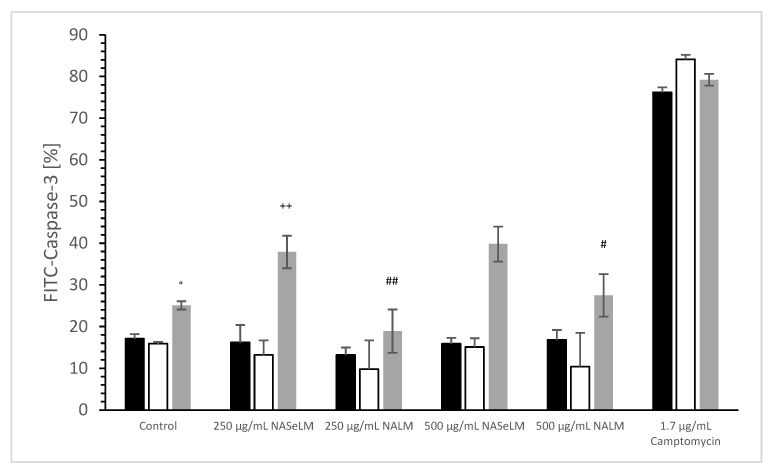
Caspase-3 measurements on Jurkat cells in absence or presence of 250 and 500 µg/mL NALM or NASeLM concentrations after 72 h on Jurkat cell compared to control or Camptomycin. ° *p* < 0.05: significance of control signal between 24 and 48 h to 72 h. ^++^ *p* < 0.01: significance between 250 and 500 µg/mL NASeLM compared to control signal after 72 h incubation. ^#^ *p* < 0.05: significance between 500 µg/mL NASeLM and 500 µg/mL NALM after 72 h incubation. ^##^ *p* < 0.01: significance between 250 µg/mL NASeLM and 250 µg/mL NALM after 72 h incubation.

No difference was estimated between 24 h or 48 h incubation of 250 and 500 µg/mL NALM or NASeLM compared to the control (incubation), as shown in Figure 3. The use of 250 and 500 µg/mL after 72 h of incubation also showed no difference to the initial control. The use of 250 and 500 µg/mL of NALM after 72 h of incubation on Jurkat cells also showed no difference to the initial control. However, 250 µg/mL NASeLM showed an increase in caspase-3 activity in Jurkat cells after 72 h (37.9 ± 3.9%; n = 3), which resulted in a significance of *p* < 0.01 compared to the control (25.1 ± 1.0%; n = 3). An increase to 500 µg/mL (39.8 ± 4.2%; n = 3) showed nearly the same amount of caspase activity as 250 µg/mL, so no significant difference was obtained.

### 3.2. MTC-SK Cell Lines

#### 3.2.1. Cell Proliferation Experiment

**Figure 4:**
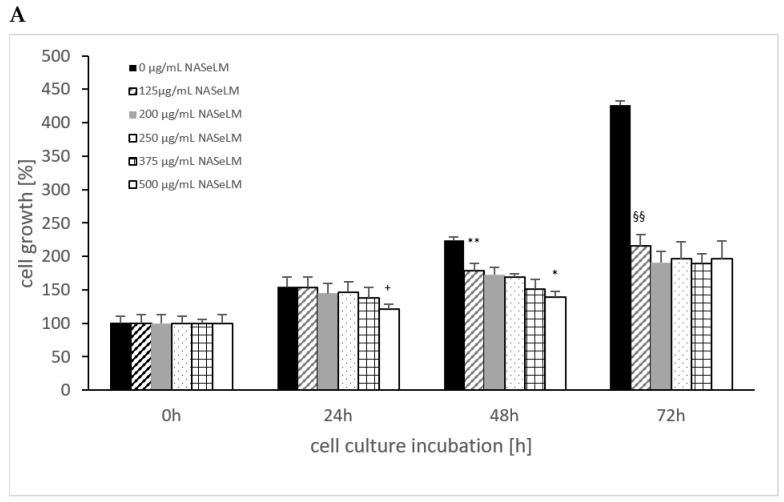

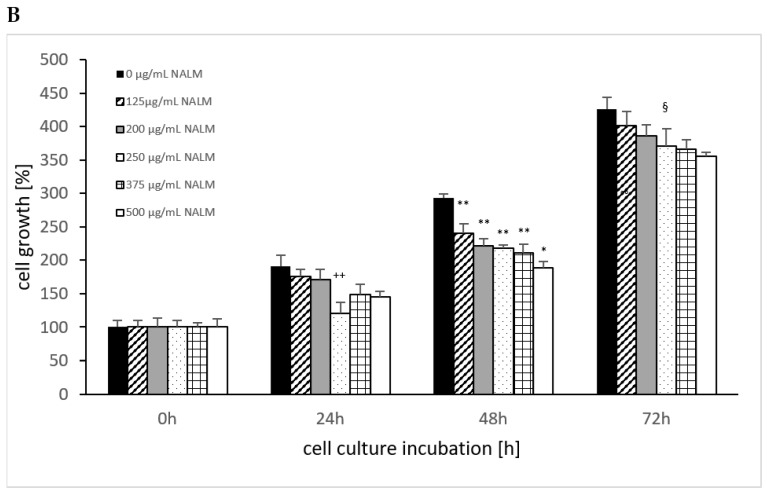
Cell growth on MTC-SK cells in absence or presence of different NASeLM (**A**) or NALM (**B**) concentrations after 24, 48, and 72 h. (**A**): ^+^ *p* < 0.05: significance of control signal compared to 250 µg/mL NASeLM after 24 h. ** *p* < 0.01: significance between control and 125 µg/mL NASeLM after 48 h incubation. * *p* < 0.05: significance between 500 µg/mL NASeLM and 125 µg/mL NASeLM after 48 h incubation. ^§§^ *p* < 0.01: significance between 250 µg/mL NASeLM and control after 72 h incubation; (**B**): ^++^ *p* < 0.01: significance between control and 250 and 500 µg/mL NALM after 24 h incubation. ** *p* < 0.01: significance between control and 125, 200, 250, and 375 µg/mL NALM after 48 h incubation. * *p* < 0.05: significance between 250 µg/mL NASeLM and 500 µg/mL NALM after 48 h incubation. ^§^ *p* < 0.05: significance between control and 250, 375, and 500 µg/mL NALM after 72 h incubation.

The cell growth of MTK-SK without the use of NALM or NASeLM showed a significant increase over time, as shown in Figure 4, from 100% up to 400%. Using NASeLM (Figure 4A), the highest concentration of 500 µg/mL (121.0 ± 7.5%; n = 3) showed a significant reduction in cell growth after an incubation time of 24 h compared to the 24 h control (154.5 ± 14.5%; *p* < 0.05). After 48 h co-incubation with NASeLM, a 125 µg/mL concentration already showed a significant decrease in the cell growth of MTK-SK cells to 178.5 ± 14.0% (n = 3) compared to the 48 h control (224.0 ± 5.0%; *p* < 0.01). A further reduction in the number of cells could only be achieved with the presence of 500 µg/mL NASeLM (139.0 ± 9.0%; n = 3) with a significance of *p* < 0.05 when compared to the 125 µg/mL NASeLM. The IC50% was determined at 550 µg/mL.

After an incubation time of 72 h, the IC50% was already achieved with 125 µg/mL NASeLM, which was highly significantly reduced compared to the control (216.0 ± 12.5% vs. 426.0 ± 6.0%; n = 3; *p* < 0.001). The subsequent concentrations of 200–500 µg/mL NASeLM did not achieve any further reduction.

In the presence of 250 µg/mL NALM (121.0 ± 16.0%; n = 3), as shown in Figure 4B, a significant reduction compared to the control was achieved after 24 h of incubation (191.0 ± 16.0%; *p* < 0.01). Concentrations of 500 µg/mL did not result in a further reduction in the number of MTK-SK cells after 24 h. After an incubation period of 48 h, a 125 µg/mL NALM concentration significantly reduced the cell count from 293.5 ± 5.5% (n = 3) to 240.0 ± 14.0% (*p* < 0.01). Although the cell count was further reduced when the NALM concentration was increased, only the use of 500 µg/mL (189.0 ± 9.8%; *p* < 0.01) was able to achieve significance compared to the 125 µg/mL sample. After 72 h of co-incubation, only one concentration of NALM, 250 µg/mL, showed a significant decrease in the control cell count from 426.0 ± 17.5% (*p* < 0.01) to 355.5 ± 5.5%.

A comparison of equal concentrations of NALM and NASeLM at 48 h incubation on MTK-SK cells (Table 3) shows highly significant differences in favor of the selenium-containing amino acid. The highest significance was achieved at 250 µg/mL, followed by 500, 375, 200, and 125 µg/mL. Increasing the incubation time to 72 h also increased the significant effect of NASeLM compared to NALM at all concentrations used.

#### 3.2.2. Mitochondrial Activity of MTC-SK Cells in Presence or Absence of NASeLM or NALM

**Figure 5:**
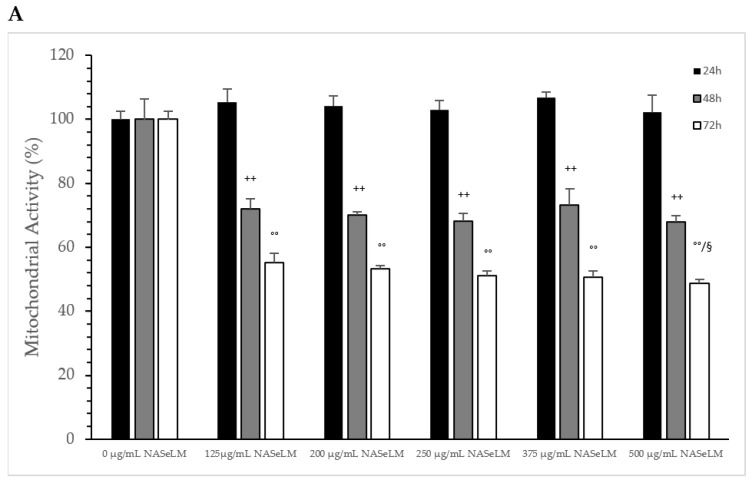

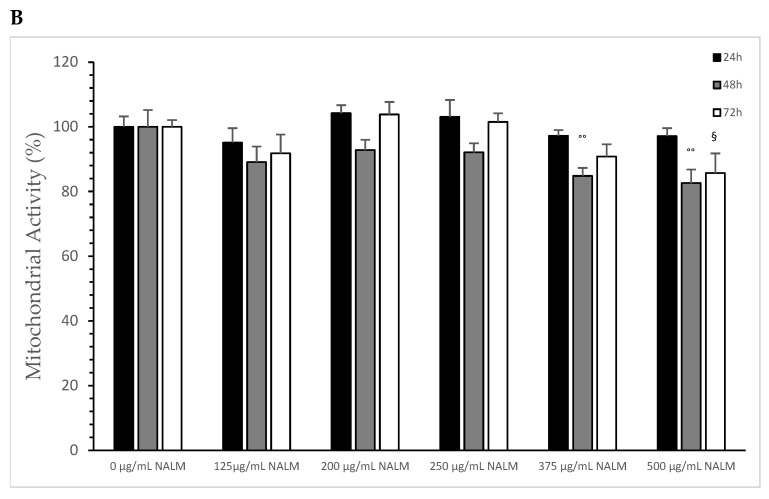
Mitochondrial activity measurements on MTC-SK cells in absence or presence of different NASeLM (**A**) or NALM (**B**) concentrations after 24, 48, and 72 h. (**A**): ^++^ *p* < 0.05: significance between control and 125, 200, 250, 375, and 500 µg/mL NASeLM after 48 h incubation. °° *p* < 0.01: significance between control and 125, 200, 250, 375, and 500 µg/mL NASeLM after 72 h incubation. ^§^ *p* < 0.05: significance between 125 µg/mL NASeLM and 500 µg/mL NASeLM after 72 h incubation; (**B**): °° *p* < 0.01: significance between control and 375 and 500 µg/mL NALM after 72 h incubation. ^§^ *p* < 0.05: significance between control and 500 µg/mL NALM after 72 h incubation.

The influence of NASeLM on the mitochondrial activity of MTS-SK cells is shown in Figure 5A, depicting that all concentrations used from 0 to 500 µg/mL after 24 h period showed did not exhibit enough influence to minimize it. After 48 h of co-incubation with 125 µg/mL NASeLM (72.1 ± 3.1%; n = 3; *p* < 0.001), there was a significant reduction in activity compared to the 48 h control (100.0 ± 6.5%). The presence of 200, 250, 375, and 500 µg/mL did not reduce the activity any further. After 72 h of incubation, there was almost a 50% reduction in mitochondrial activity at 125 µg/mL, with the calculated IC50% corresponding to a concentration of 200 µg/mL. The highest concentration of 500 µg/mL (48.7 ± 1.2%; n = 3; *p* < 0.05) also achieved a further significant decrease compared to the 125 µg/mL sample.

NALM exhibited a small effect on the mitochondrial activity of MTC-SK cells, as shown in Figure 5B, and only at high concentrations and a longer incubation time: 375 µg/mL NALM decreased the activity from 100 ± 5.2% to 84.8 ± 2.5% (n = 3) after 48 h with a significance of *p* < 0.05. 500 µg/mL NALM did not cause any further change in activity compared to the 375 µg/mL NALM sample. At 72 h of incubation, only the highest concentration of 500 µg/mL (85.7 ± 6.1%; n = 3) was able to significantly reduce the mitochondrial activity relative to the 72 h control (100 ± 2.1%; *p* < 0.05).

Table 4 shows the comparison of mitochondrial activity between NASeLM and NALM at the same concentrations after 48 and 72 h of incubation on MTC-SK cells. After 48 h, 125 µg/mL NASeLM showed a significantly better reduction compared to the NALM sample at *p* = 0.005, 200 µg/mL NASeLM at *p* = 0.003, 250 µg/mL NASeLM at *p* = 0.0004, 375 µg/mL NASeLM at *p* < 0.025, and 500 µg/mL NASeLM at *p* = 0.005, respectively, compared to the same concentration of NALM. Mitochondrial activity decreased proportionally with increasing incubation time to 72 h: here, all used concentrations of NASeLM showed a highly significant decrease in mitochondrial activity compared to the same NALM concentrations of up to *p* < 0.0001.

#### 3.2.3. Caspase-3 Activated Apoptosis Measurements of MTC-SK Lines in Presence or Absence of NASeLM or NALM

**Figure 6:**
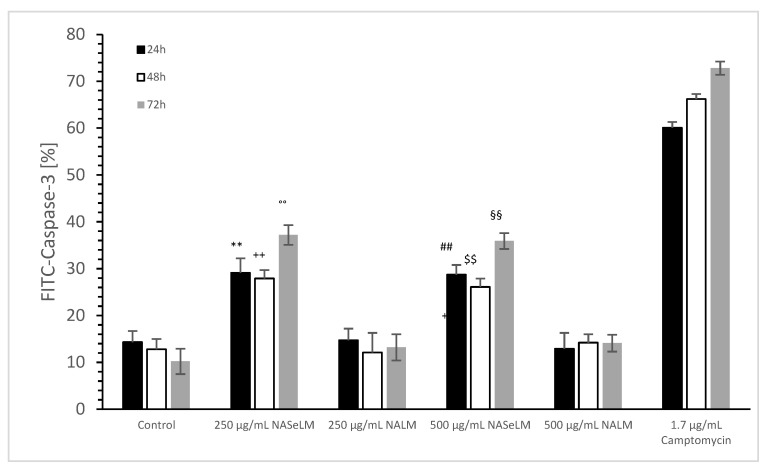
Caspase-3 measurements on MTC-SK cells in absence or presence of 250 and 500 µg/mL NALM or NASeLM concentrations after 72 h on Jurkat cell compared to control or Camptomycin. ** *p* < 0.01: significance of 250 µg/mL NASeLM compared to control or 250 µg/mL NALM after 24 h incubation. ^++^ *p* < 0.01: significance of 250 µg/mL NASeLM compared to control or 250 µg/mL NALM after 72 h incubation. °° *p* < 0.01: significance between 250 µg/mL NASeLM compared to control or 250 µg/mL NALM signal after 72 h incubation. ^##^ *p* < 0.01: significance of 250 µg/mL NASeLM compared to control or 250 µg/mL NALM after 24 h incubation. ^$$^ *p* < 0.01: significance of 250 µg/mL NASeLM compared to control or 250 µg/mL NALM after 72 h incubation. ^§§^ *p* < 0.01: significance between 250 µg/mL NASeLM compared to control or 250 µg/mL NALM signal after 72 h incubation.

The caspase-3 activity of NALM and NASeLM was compared to a negative control and a positive control (Camptomycin) after 24 h, 48 h, and 72 h of incubation on MTC-SK cells, as shown in Figure 6. NALM showed no increase in caspase-3 activity, neither at concentrations of 250 and 500 µg/mL nor with different incubation times. It remained almost the same as the respective baseline controls. NASeLM showed a caspase-3 activity of 29.1 ± 3.1% (n = 3) already after 24 h with a concentration of 250 µg/mL and was significantly higher than the 24 h baseline control (14.3 ± 2.4%; n = 3; *p* < 0.01) and the 24 h 250 µg/mL NALM sample (14.7 ± 2.5%; n = 3; *p* < 0.01). 

However, increasing the NASeLM concentration to 500 µg/mL (35.9 ± 1.7%; n = 3) failed to induce a further increase in caspase-3 activity, again maintaining a significance of *p* < 0.01 compared to the negative control and the 500 µg/mL NALM sample (14.2 ± 1.8%; n = 3). After 48 h, 250 µg/mL (27.9 ± 1.8%; n = 3) and 500 µg/mL NASeLM (26.1 ± 1.8%; n = 3) were equally effective in increasing caspase activity in the MTC-SK cells, but did not differ. Similar to the mitochondrial activity at 24 h of incubation, both showed a clear significance to their equivalent NALM samples (250 µg/mL: 12.1 ± 2.8%; n = 3; *p* < 0.01. 500 µg/mL: 14.2 ± 1.8%; n = 3; *p* < 0.01), as well as to the initial control.

After an incubation period of 72 h, there was a further increase in caspase-3 activity at 250 µg/mL (37.2 ± 2.1%; n = 3) compared to the 24 h and 48 h NASeLM samples (*p* < 0.01). The same was achieved with the 500 µg/mL NASeLM sample (35.9 ± 1.7%; n = 3; *p* < 0.01) compared to the negative control (10.2 ± 2.7%; n = 3; *p* < 0.01) or 500 µg/mL NALM sample (13.2 ± 2.8%; n = 3; *p* < 0.01) after 72 h of incubation, which, however, did not differ significantly from the 250 µg/mL NASeLM sample.

## 4. Discussion

MTC-SK and Jurkat cells have one thing in common, they both have a high carbonyl protein content in their cell membrane, which is due to a high radical metabolism, as we have shown. If this is reduced by substances, like 2-oxoglutarate and/or 5-hydroxy-methyl-furfurale, both cell lines appear to lead to apoptosis via mitochondrial pathways. For this reason, both cell lines were selected to see whether the addition of NALM or NASeLM can also influence the metabolism in cancer cells with high radical formations or whether selenium plays a greater role here in contrast to the non-selenium-containing amino acid methionine.

It is known from a meta-analysis or meta-regression that high selenium exposure has a protective effect on the risk of the onset of cancer and that a high selenium level in plasma or serum even prevents cancer. This particularly applies to cancers such as breast cancer, lung cancer, esophageal cancer, stomach, and pancreatic cancer. Colorectal cancer, bladder cancer, and skin cancer showed no cancer prevention with increased selenium levels [6].

Recent clinical studies were able to establish a link between selenium levels and papillary thyroid cancer in a Chinese population, with selenium levels being lower in females than in males [4,16]. The study thus establishes a connection that a high serum level of selenium can be a protective factor in papillary thyroid cancer. We used MTC-SK cells, a medullary thyroid cancer cell line, and tested the behavior of NASeLM and the non-selenium-containing amino acid NALM. It turned out that NASeLM clearly showed a higher caspase-3 activity than NALM and forced this type of cancer into apoptosis. This occurred because both the cell growth and mitochondrial activity of medullary thyroid carcinoma were also influenced by NASeLM in a time-dependent manner to a marked reduction. In terms of concentration, 125 µg/mL of NASeLM at 72 h of incubation was sufficient to reduce cell growth and mitochondrial activity by 50% after 72 h of incubation. Even 250 µg/mL NASeLM already indicated a maximum value of caspase-3 activity during apoptosis, which could no longer be surpassed when the concentration was increased up to 500 µg/mL. NALM was also able to reduce mitochondrial activity and cell growth, but to a much lesser extent than NASeLM. NALM was not able to influence caspase activity in any way and thus did not lead to cell death in the MTC-SK cells. This is in contradiction to several previous studies, in which the restriction of methionine significantly reduced cell growth in cancer cells [17,18]. In these studies, however, folic acid- and cyanocobalamin-rich media were used, in which methionine was added or left out. Only the failure to increase caspase-3 activity was confirmed with our study when NALM was used. It can therefore be speculated that methionine alone may reduce cell growth and mitochondrial activity by other mechanisms. A more recent study, on the other hand, shows an impairment of the growth rate of liver cancer cells, which occurs via significantly increased AMP-activated protein kinase (AMPK) inhibition and the mTOR signaling pathway, which could coincide with our results on MTC-SK cells [19].

Therefore, in this work, we also collected data from several NALM concentrations on another type of cancer cells, namely leukemia cells, in the form of the Jurkat cell line. Here, in contrast to the results on MTC-SK cells, NALM and NASeLM both minimized mitochondrial activity and cell growth, but there was no difference between methionine and the selenium-containing methionine substance. It is assumed that the effect on Jurkat cells comes from methionine rather than selenium. If one compares the efficiency of NALM in both cell lines, it appears to be the same. However, in contrast to NALM, NASeLM shows caspase-3 activity on Jurkat cells at a similar level as on MTC-SK cells. The effect of NALM in both cell lines does not exhibit any effect. It is shown that the selenium-containing amino acid NASeLM strongly reduces cell growth and mitochondrial activity in MTC-SK cells, like in Jurkat cells, via an additional mechanism of action, which is not dependent on radical-induced metabolism—as measured by carbonyl protein content [13,14].

It is shown that the selenium-containing amino acid NASeLM strongly reduces cell growth and mitochondrial activity in MTC-SK cells in comparison to Jurkat cells via an additional mechanism of action, which is not dependent on radical-induced metabolism—as measured by carbonyl protein content. Presumably, in MTC-SK cells, the added selenium in the form of NASeLM can be converted into other selenium-containing forms (such as selenium-containing proteins) that only occur in thyroid cancer cells (such as iodothyronine deiodase or thioreduxin reductase) as described in a paper by Venture et al. [20]. In their paper, they surveyed all scientific studies on selenium and thyroid diseases and were able to show that organic selenium-containing substances, in particular, as well as NASeLM, displayed a beneficial health effect on thyroid diseases and on poorly differentiated thyroid cancer [21]. Here, another possible mechanism of action that is pointed out is that seleno-methionine inhibits cell growth in a dose-dependent manner via the LncRNA-NONMMUT014201/miR-6963-5p/Srprb pathway. Moreover, NASeLM could be a potential drug for the treatment of poorly differentiated thyroid cancer.

## 5. Conclusions

Our data showed that NALM has an inhibitory cell growth effect and mitochondrial-deactivating effect on Jurkat and MTC-SK cells. However, caspase-3 activity was not activated. Furthermore, NASeLM, the selenium-containing form of NALM, showed similar results on Jurkat cells, with caspase-3 activity being increased. On MTC-SK cells, NASeLM not only displayed caspase-3 activity but was also able to significantly reduce cell growth and mitochondrial activity, more than NALM. We will carry out further studies to confirm that other mechanisms may also be involved in the effects that NALM and NaSeLM exhibit.

## Figures and Tables

**Table 1:** Comparison of cell growth (%) in absence or presence of NASeLM and NALM at same concentrations used between 125 and 500 µg/mL after 48 h or 72 h incubation on Jurkat cells. ns = no significance.

Cell Growth	48 h	*p*	72 h
	%		%
125 µg/mL NASeLM	121.0 +/− 12.5	ns	204.0 +/− 41.0
125 µg/mL NALM	139.4 +/− 33.5		204.0 +/− 46.0
200 µg/mL NASeLM	131.0 +/− 7.0	ns	196.0 +/− 33.5
200 µg/mL NALM	93.5 +/− 28.0		181.5 +/− 66.0
250 µg/mL NASeLM	119.0 +/− 4.5	ns	169.0 +/− 25.5
250 µg/mL NALM	109.0 +/− 29.0		104.5 +/− 41.0
375 µg/mL NASeLM	102.5 +/− 6.0	ns	131.0 +/− 21.0
375 µg/mL NALM	114.0 +/− 21.0		106.0 +/− 31.0
500 µg/mL NASeLM	113.5 +/− 9.0	ns	119.0 +/− 27.5
500 µg/mL NALM	110.5 +/− 7.5		104.0 +/− 35.5

**Table 2:** Comparison of the mitochondrial activity of Jurkat cells in absence or presence of NASeLM and NALM at same concentrations used between 125 and 500 µg/mL after 48 h or 72 h incubation. ns = no significance.

Mitochondrial Activity	48 h	*p*	72 h	*p*
	%		%	
125 µg/mL NASeLM	78.2 +/− 8.4	ns	67.9 +/− 4.2	ns
125 µg/mL NALM	78.0 +/− 6.2		58.9 +/− 8.4	
200 µg/mL NASeLM	58.7 +/− 6.2	ns	43.1 +/− 2.8	ns
200 µg/mL NALM	56.9 +/− 5.9		43.1 +/− 5.2	
250 µg/mL NASeLM	62.4 +/− 7.1	ns	55.2 +/− 8.2	ns
250 µg/mL NALM	59.9 +/− 4.9		48.9 +/− 6.7	
375 µg/mL NASeLM	50.7 +/− 4.9	ns	37.9 +/− 1.8	ns
375 µg/mL NALM	50.8 +/− 4.8		36.1 +/− 1.2	
500 µg/mL NASeLM	46.8 +/− 11.2	ns	36.0 +/− 12.2	ns
500 µg/mL NALM	43.9 +/− 2.0		31.8 +/− 2.9	

**Table 3:** Comparison of cell growth (%) in absence or presence of NASeLM and NALM at same concentrations used between 125 and 500 µg/mL after 48 h or 72 h incubation on MTC-SK cells.

Cell Growth	48 h	*p*	72 h	*p*
	%		%	
125 µg/mL NASeLM	178.5 +/− 14.0	0.02	216.0 +/− 12.5	<0.001
125 µg/mL NALM	240.5 +/− 14.0		401.0 +/− 21.0	
200 µg/mL NASeLM	172.5 +/− 11.0	0.009	191.0 +/− 17.0	<0.001
200 µg/mL NALM	221.0 +/− 16.0		386.0 +/− 17.1	
250 µg/mL NASeLM	169.0 +/− 4.5	<0.001	196.0 +/− 20.1	<0.001
250 µg/mL NALM	218.0 +/− 4.1		371.0 +/− 18.5	
375 µg/mL NASeLM	151.0 +/− 14.0	0.005	189.0 +/− 14.5	<0.001
375 µg/mL NALM	210.5 +/− 14.2		366.0 +/− 13.9	
500 µg/mL NASeLM	139.0 +/− 9.0	0.002	196.0 +/− 27.5	<0.001
500 µg/mL NALM	189.0 +/− 9.8		355.5 +/− 5.5	

**Table 4:** Comparison of the mitochondrial activity of MTC-SK cells in absence or presence of NASeLM and NALM at same concentrations used between 125 and 500 µg/mL after 48 h or 72 h incubation.

Mitochondrial Activity	48 h	*p*	72 h	*p*
	%		%	
125 µg/mL NASeLM	72.1 +/− 2.1	0.005	55.2 +/− 3.0	0.0006
125 µg/mL NALM	89.1 +/− 4.8		91.8 +/− 5.8	
200 µg/mL NASeLM	70.1 +/− 1.1	0.003	53.2 +/− 1.0	<0.0001
200 µg/mL NALM	92.8 +/− 3.2		103.8 +/− 3.9	
250 µg/mL NASeLM	68.1 +/− 2.6	0.0004	51.2 +/− 1.3	<0.0001
250 µg/mL NALM	92.1 +/− 2.8		101.5 +/− 2.7	
375 µg/mL NASeLM	73.2 +/− 5.2	0.025	50.7 +/− 1.9	<0.0001
375 µg/mL NALM	84.8 +/− 2.5		90.8 +/− 3.8	
500 µg/mL NASeLM	68.0 +/− 1.8	0.005	48.7 +/− 1.2	0.0005
500 µg/mL NALM	82.6 +/− 4.2		85.7 +/− 6.1	

## Data Availability

The data presented in this study are available in this article.
